# Topographic organization of the human caudate functional connectivity and age-related changes with resting-state fMRI

**DOI:** 10.3389/fnsys.2022.966433

**Published:** 2022-09-23

**Authors:** Jonathan F. O'Rawe, Hoi-Chung Leung

**Affiliations:** ^1^Integrative Neuroscience Program, Department of Psychology, Stony Brook University, Stony Brook, NY, United States; ^2^National Institute of Mental Health Intramural Program, National Institutes of Health, Bethesda, MD, United States

**Keywords:** cortico-striatal circuits, basal ganglia, functional mapping, topography, aging, flexible behavior, executive functions, fMRI

## Abstract

The striatum is postulated to play a central role in gating cortical processing during goal-oriented behavior. While many human neuroimaging studies have treated the striatum as an undivided whole or several homogeneous compartments, some recent studies showed that its circuitry is topographically organized and has more complex relations with the cortical networks than previously assumed. Here, we took a gradient functional connectivity mapping approach that utilizes the entire anatomical space of the caudate nucleus to examine the organization of its functional relationship with the rest of the brain and how its topographic mapping changes with age. We defined the topography of the caudate functional connectivity using three publicly available resting-state fMRI datasets. We replicated and extended previous findings. First, we found two stable gradients of caudate connectivity patterns along its medial-lateral (M-L) and anterior-posterior (A-P) axes, supporting findings from previous tract-tracing studies of non-human primates that there are at least two main organizational principles within the caudate nucleus. Second, unlike previous emphasis of the A-P topology, we showed that the differential connectivity patterns along the M-L gradient of caudate are more clearly organized with the large-scale neural networks; such that brain networks associated with internal vs. external orienting behavior are respectively more closely linked to the medial vs. lateral extent of the caudate. Third, the caudate's M-L organization showed greater age-related reduction in integrity, which was further associated with age-related changes in behavioral measures of executive functions. In sum, our analysis confirmed a sometimes overlooked M-L functional connectivity gradient within the caudate nucleus, with its lateral longitudinal zone more closely linked to the frontoparietal cortical circuits and age-related changes in cognitive control. These findings provide a more precise mapping of the human caudate functional connectivity, both in terms of the gradient organization with cortical networks and age-related changes in such organization.

## Introduction

The primate striatum is composed of the putamen and the caudate nucleus, which are the main input nuclei of the basal ganglia in the cortico-striatal dopaminergic circuitry (Alexander et al., 1986; Middleton and Strick, 2000; Haber, 2003). Clinical and basic neuroscience research studies have found a critical role of the striatum in gating, a unique function of selecting action plans to optimize goal attainment (Mink, 1996; O'Reilly, 2006; Grahn et al., 2008; Frank and Badre, 2012; Chatham et al., 2014; Provost et al., 2015 for a more recent review). This is evident in classic movement disorders such as Parkinson's Disease (PD) and Huntington's Disease (HD), both show pathology involving the striatum and express symptoms stemming from dysfunctional motor gating (Bernheimer et al., 1973). It is further recognized that individuals with PD and HD also demonstrate various cognitive symptoms, suggesting a much more complex outcome of striatal dysfunctions (Brandt et al., 1988; Cooper et al., 1991; Lawrence et al., 1998; Janvin et al., 2006). In particular, the caudate nucleus is closely linked to cognitive control or executive functions (Levy et al., 1997; Lewis et al., 2003; Robinson et al., 2012), with recent studies showing alterations in caudate circuitry can greatly impact cognitive functioning in the aging human brain (Huang et al., 2017; Rieckmann et al., 2018). While previous neuroimaging studies have repeatedly demonstrated that the human striatum can be parcellated into multiple functional subdivisions (e.g., Barnes et al., 2010; Choi et al., 2012; Jung et al., 2014; Janssen et al., 2015; Jarbo and Verstynen, 2015), it remains an open question on how the caudate circuits organize functionally and how such organization changes with age and cognitive decline.

Understanding the organization of the caudate nucleus is a critical step toward understanding the computations necessary to gate goal directed behavior (Thivierge and Marcus, 2007; Jbabdi et al., 2013). Systematic neuroanatomical tracing studies in non-human primates suggested that the connections between the neocortex and the caudate are extensive and systematically organized. Early anatomical findings using silver impregnation of degenerating axons following systematic lesioning of the cortex suggested that cortical projections to the caudate were fairly restricted along the rostral-caudal axis, such that more caudal cortex projects to more caudal caudate and vice versa for the rostral projections (Carman et al., 1965; Webster, 1965; Kemp and Powell, 1970). It has been suggested that this rostral-caudal anatomical arrangement of caudate is systematically linked to the anterior-posterior frontal cortical areas and their corresponding implications in affective, cognitive, and motor processes (Haber, 2003). However, it has also been shown that the anterograde tracing from the cortex exhibit far less restriction along the longitudinal axis, with several studies showing a consistent organization across the medial-lateral and the dorsal-ventral axes (Selemon and Goldman-Rakic, 1985; Ferry et al., 2000). It was further demonstrated that that areas which share cortico-cortical connections also show colocalization in projections to the caudate (Yeterian and Van Hoesen, 1978; Selemon and Goldman-Rakic, 1985; Parthasarathy et al., 1992).

In human neuroimaging studies, the multiple parallel organization of cortico-striatal loops has been demonstrated initially using seed-based functional connectivity analysis of resting-state fMRI data (Di Martino et al., 2008; Manza et al., 2015) and meta-analytic modeling of task-related activations (Postuma and Dagher, 2006; Robinson et al., 2012). More recent studies used supervised and unsupervised clustering methods with resting-state functional connectivity data to parcellate the striatum into multiple functional subdivisions using their correlated connectivity patterns (Barnes et al., 2010; Choi et al., 2012; Jung et al., 2014; Janssen et al., 2015; Jaspers et al., 2017). Different striatal parcels appeared to be associated with different canonical cortical networks (Choi et al., 2012; Jung et al., 2014) and linked to the different cognitive and sensorimotor functions implicated by these networks (Yeo et al., 2011). While most of previous studies have revealed the global anterior-posterior organization, the medial-lateral organization is often being overlooked or less emphasized (but see Choi et al., 2012; Jarbo and Verstynen, 2015). It has also been shown that the caudate nucleus exhibits more complex functional relationship with the cortical association areas with converging zones from disparate frontal and parietal areas (Jarbo and Verstynen, 2015; Choi et al., 2017). While behavioral validation is scarce in previous studies of functional connectivity profiles, differential behavioral functions have been revealed in examination of coactivation data reported in the literature (Pauli et al., 2016).

Most recent human resting-state functional connectivity studies have used voxel-based clustering approaches, which derived about 3–9 caudate parcels at the most (e.g., Jung et al., 2014; Janssen et al., 2015) and have rarely taken advantage of the continuous nature of the sampling space of fMRI data. Animal models, however, suggested a consistent topographic relationship of frontal cortex projections into the striatum has been shown along a diagonal path from dorsolateral to ventromedial striatum (Haber, 2003). That is, more motor-related processes, such as those from precentral sulcus, project to dorsolateral areas of the striatum, while higher order control and reward processes, such as those implemented by middle frontal gyrus and ventromedial prefrontal cortex, project more rostrally to the ventromedial striatum (Haber et al., 2000). To delineate continuous forms of topographic organization in the human brain, recent approach utilized gradient techniques and/or by using space as a factor of analysis (Jarbo and Verstynen, 2015; Haak et al., 2017; Marquand et al., 2017; O'Rawe et al., 2019). Thus, unlike the conventional approaches, the gradient approach avoids averaging voxels within a heterogeneous structure and affords mapping the structure's functional topography across the anatomical space. The topographic mapping of the striatum has been first replicated in humans using resting-state functional connectivity together with the gradient method that examined across the entire striatal space (Marquand et al., 2017). Another study using a different gradient method further showed the existence of a medial-lateral organizational scheme in the caudate and that is not evident in the putamen (O'Rawe et al., 2019). Thus, it seems necessary to further examine the organization of the caudate's functional connectivity separately instead of across the entire striatum as well as how the caudate circuits map with the cortical networks.

The gradient methods with continuous sampling of data across space not only allow using the space as a continuous factor but also afford simultaneous estimates of various organizational schemes within the same brain structure of the same individual. In this study, we used space as a factor to examine linearly dependent changes in caudate functional connectivity across its anatomical space using resting-state fMRI data from three publicly available databases. We conducted analyses to identify and characterize at least the three potential orthogonal organizations: medial-lateral (M-L), anterior-posterior (A-P), or dorsal-ventral (D-V). We expected to find two gradients of functional organization across the caudate's anatomical space, A-P and M-L. Since reduction in both striatal and midbrain dopamine was associated with a reduction in executive functions or cognitive control of behavior in healthy aging including larger switch costs (Berry et al., 2016), lowered processing speed (Bäckman et al., 2000) and altered reward processing (Dreher et al., 2008), we further explored the effects of aging on the caudate's spatial organization and its relationship with age-related changes in cognitive control. As recent work has demonstrated that dopamine signals entering the striatum are spatially biased and sweep along the M-L direction (Hamid et al., 2021), we expected to find evidence of an aging-related reduction in caudate spatial organization (particularly along the M-L axis) due to a loss of this spatially biased signal and a decrease in the integrity of caudate spatial organization would be related to age related decreases in cognitive performance.

## Materials and methods

### Image data and acquisition parameters

For the primary caudate topography analyses, we utilized the Cambridge Buckner sample of 198 subjects (123 female from the 1000 Connectomes Project (Biswal et al., 2010) (http://fcon_1000.projects.nitrc.org), ages 18-30 (*M* = 21.03, *SD* = 2.31). For replication and age-related analyses, we utilized the 207 subjects (87 female) in the Rockland dataset (http://fcon_1000.projects.nitrc.org/indi/pro/nki.html), ages 4–85 (*M* = 35.00, *SD* = 20.00) and the first (in order of release) 377 subjects (238 female) of the Rockland Enhanced dataset, ages 8–85 (*M* = 42.11, *SD* = 20.34). Image data of all subjects from the Cambridge Buckner database passed the screening for motion and other image artifacts (with at least 2/3 usable data per subject). For the Rockland dataset, 18 subjects were excluded, with the remaining 189 subjects (78 female), ages 4–85 (*M* = 35.70, *SD* = 19.89) used in the final analysis. For the Rockland enhanced dataset, 78 subjects were excluded, leaving 299 subjects (194 female), ages 8–85 (*M* = 40.96, *SD* = 20.33).

The Cambridge Buckner data (Siemens 3T Trim Trio): T1-weighted structural images were collected using MPRAGE with the following image parameters: slices = 192, matrix size = 144 × 192, voxel resolution = 1.20 × 1.00 × 1.33 mm^3^. Resting state functional images were acquired using an EPI sequence with the following parameters: 47 interleaved axial slices, TR = 3,000 ms, TE = 30 ms, flip angle = 85 deg, voxel resolution = 3 × 3 × 3 mm^3^ (119 volumes).

The NKI/Rockland data (Siemens 3T Trim Trio): T1-weighted structural images were collected using MPRAGE with the following parameters: slices = 192, matrix size = 256 × 256, resolution = 1 × 1 × 1 mm^3^. Resting state functional images were acquired using an EPI sequence with the following parameters: 38 interleaved axial slices with gap = 0.33 mm, TR = 2,500 ms, TE = 30 ms, flip angle = 80 deg, voxel resolution = 3 × 3 × 3 mm^3^ (260 volumes).

Enhanced Rockland (Siemens 3T Trim Trio): T1-weighted images were collected using MPRAGE with the following parameters: slices = 176, matrix size = 250 × 250, resolution = 1 × 1 × 1 mm^3^. Resting state functional images were acquired with the following parameters: 64 interleaved axial slices with 0 gap, multi-band acceleration factor = 4, TR = 1,400 ms, TE = 30 ms, flip angle = 65 deg, voxel resolution = 2 × 2 × 2 mm^3^ (404 volumes in total).

### Image preprocessing

Prior to analysis images were preprocessed utilizing SPM12 (http://www.fil.ion.ucl.ac.uk/spm/software/spm12/). Images were first corrected for slice timing, and then realigned to the middle volume according to a 6-parameter rigid body transformation. Structural images were coregistered with the mean functional image, segmented, and then normalized to the MNI template using both linear and non-linear transformations. Functional images were then normalized utilizing the same parameters as the structural normalization. No explicit smoothing was applied to the functional images during any of these steps.

Minor head motion and noise were addressed by applying additional preprocessing steps, using either CONN (http://www.alfnie.com/software/conn) or custom matlab scripts. A nuisance regression was constructed to regress out the following confounding variables: 6 motion parameters up to their second derivatives, scans with excessive head motion (framewise displacement > 0.5 mm), effects of session onset, modeled physiological signal generated through aCompCor (Behzadi et al., 2007) of the white matter and CSF voxels, and a linear drift component. For the Cambridge Buckner and Rockland datasets, the residuals of this regression were filtered using a bandpass filter (0.008 < *f* < 0.09), while for the Rockland Enhanced dataset this step was done simultaneously with regression (Hallquist et al., 2013). Finally, the residuals were despiked using a tangent squashing function.

### Spatially dependent gradient functional connectivity pipeline

We named the pipeline Fine Relational Spatial Topography (FiRST). The goal of FiRST is to systematically explore spatial biases in functional connectivity of a region of interest. In this study, we asked whether certain areas of the brain are more strongly functionally coupled to the medial extent of the caudate in comparison to the lateral caudate, and similarly for the anterior-posterior and dorsal-ventral dimensions. To do this, we constructed a multiple regression to predict the caudate's voxel-wise connectivity with the rest of the brain using a Euclidean description of the space parameters for each of voxel in the caudate. If the strength of connectivity for a particular brain voxel changed linearly along a spatial dimension (M-L, A-P, or D-V) of the caudate, then the model would produce a larger coefficient for that dimension for that brain voxel. We chose this linear gradient approach because it represents one of the simplest methods to explore spatial dependencies in functional connectivity and provides a robust framework to explore brain network and brain-behavior relationships.

The caudate mask includes the entire structure from head to tail, using the automated anatomical labeling atlas (Tzourio-Mazoyer et al., 2002), with the region of interest aligned and resampled to the space of each data sample. In order to reduce error from any minor misalignment across subjects, we eroded the first outermost layer of voxels from the caudate mask.

**Figure 1:**
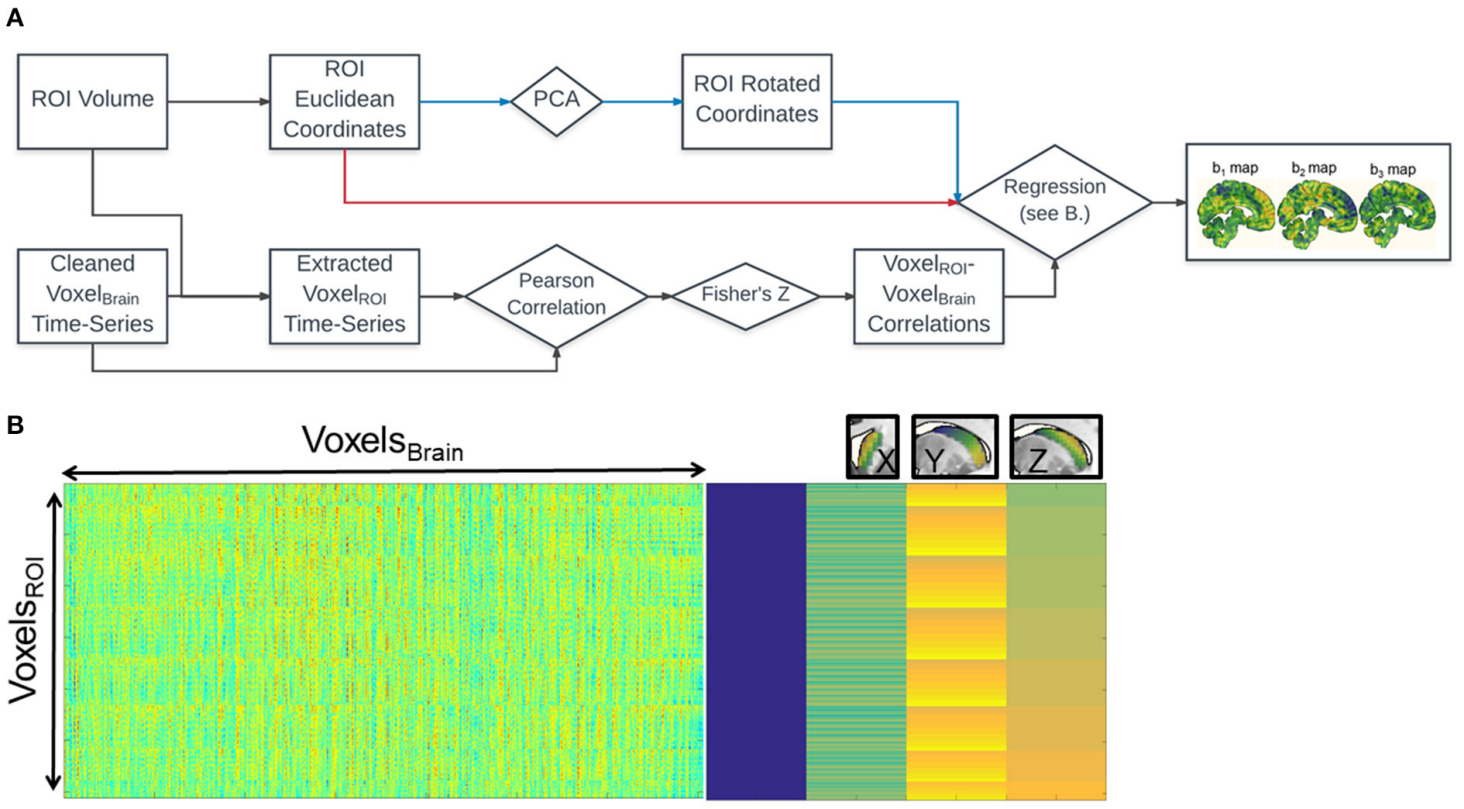
Fine Relational Spatial Topography (FiRST) approach. **(A)** Analysis pipeline. Squares denote measurements and diamonds denote operations. The voxel wise time-series from the entire brain (Voxel_Brain_) are correlated with the voxel wise time-series of the ROI (Voxel_ROI_). The resultant *k* × *n* matrix (where *k* = number of voxels in the ROI, and *n* = number of voxels in the brain) is then regressed against the x, y, z coordinate space of the ROI, either in Euclidean coordinates or their corresponding rotated values derived from PCA (red or blue arrows in the flowchart). This regression produces 3 beta maps, denoting preference in connectivity along a particular directional gradient along the ROI (b_1_ for M-L, b_2_ for A-P, b_3_ for D-V). **(B)** The visualization of the design matrix for the regression analysis. The rightmost side is equivalent to the conventional SPM design matrix for the ROI's x, y, z coordinates, while the leftmost side is the input into the regression. Right above the design matrix illustrates the three orthogonal caudate gradients.

Figure 1 shows the schematic diagram for the steps used in the analysis. For each voxel within the caudate, we calculated its functional connectivity with the rest of the voxels in the brain (*via* Pearson correlation of the timeseries). The correlation values of each voxel in the whole brain were vectorized and then transformed with Fisher's Z transformation to reduce skewness due to bounded quantities in the resulting connectivity maps (Figure 1A). For each voxel in the brain, we then fit a linear regression (standardized ordinary least squares) of that voxel's Fisher's *Z*-values with the coordinates of each of the seed voxels (Figure 1B).

This process generates three β-weight maps representing change in connectivity strength (Fisher's *Z-*value) along each of the three Euclidean dimensions (x, y, and z) of the caudate for each voxel in the brain (Figure 1A, right). That is, three spatial gradient connectivity maps (M-L, A-P, and D-V) for the caudate in each hemisphere. Note, prior to this regression step we orthogonalized caudate's coordinate space utilizing Principal Component Analysis (PCA), a form of whitening. A rotation of any object without perfect symmetry within Euclidean space would induce a correlation between its coordinate space and a loss of spatial variance when performing regression. Orthogonalizing the space prior to regression can recover the spatial variance, though the dimensions may no longer follow traditional axes, depending on the extent of the rotation and the anatomical literature's definitions. The FiRST pipeline has been released and made available *via* the NITRC platform (https://www.nitrc.org/projects/first/).

Second level *t*-tests were then conducted to generate group level gradient connectivity maps for each dataset. We further calculated disjunction maps across the second level gradient maps in order to select voxels that demonstrated a strict bias across only one of the dimensions (e.g., a voxel has a significant (α = 0.001) negative beta in the M-L axis but is no different than zero in either the A-P or D-V axes). We further restricted the final gradient maps to voxels that were above threshold in 2/3 of the datasets analyzed (“replication maps”).

To examine the relationship between the caudate gradients and the cortical areas, we quantified the correlation between each caudate gradient and the large-scale cortical networks commonly identified in resting-state fMRI studies. Specifically, the distributed regions of 7 major cortical networks were defined according to Yeo et al. (2011), including the default, frontoparietal, dorsal attention, ventral attention, visual, somatomotor, and limbic networks. These networks are selected for comparison with similar studies in the literature (e.g., see Figure 6 in Choi et al., 2012).

### Age regression analysis

For each subject, a gradient β-weight map was constructed for each orthogonal dimension using the method described above. To test if the prototypical organization of cortical connectivity with the caudate degrades across age (i.e., gradient β-weights trending toward zero), we extracted β-weights for age from the significant voxels on each side of the second level gradient disjunction map. If we are observing an overall degradation of organization, then the positive side will have a net negative relationship with age, while the negative side will have a net positive relationship with age.

### Estimations of individual gradient integrity

In order to more directly estimation the integrity of each gradient, we used a set of techniques that are developed based our prior gradient decomposition work in the striatum (O'Rawe et al., 2019). We previously found two predominant gradients of organization in the caudate, a M-L organization that resembles the othogonalized M-L dimensions here, and a dorsolateral to ventromedial diagonal gradient that most resembles the orthogonalized A-P axis here. From this prior work, we used the discovered gradients as gradient templates. First, to demonstrate the similarity of the results using the orthogonalized coordinate system and those using the prior templates, we examined the whole brain relationship of connectivity along each of these gradient templates. To do this, we ran a voxelwise regression (same as described above for the FiRST pipeline) with the templates as regressors instead of orthogonalized coordinates. The results are highly similar, as shown by the whole brain maps displayed in Supplementary Figure S1. Then, to construct an integrity measure, we fit each subject's estimated gradient maps (estimated using non-metric multidimensional scaling) to the two gradient templates using a multivariate multiple regression:


(1) $$\left[\overset{\wedge }{i_{1},i_{2}}\right]=\left[\begin{array}{c} G_{1}\\ G_{2} \end{array}\right]\left[\begin{array}{c} \beta_{11}\;\beta_{21}\\ \beta_{12}\;\beta_{22} \end{array}\right]$$

We then took the maximum absolute value beta for the fit of each gradient template as a measure the gradient integrity for that individual, with the intuition from our previous work that each estimated space more often represents a single gradient.


(2) $$\begin{array}{l} max|\beta |=\max\left(\left|\left[\begin{array}{c} \beta_{11}\;\beta_{21}\\ \beta_{12}\;\beta_{22} \end{array}\right]\right|\right) \end{array}$$

We also constructed an alternative metric without this assumption, the sum of the absolute value beta fit of each gradient template. The two metrics correlated strongly with each other, and the results of all later tests were virtually the same using either metric (we therefore only showed results from max metric).

### Testing the relationship between caudate gradient integrity and age-related changes in relationship between caudate gradient integrity and executive function

We used Partial-Least Squares (PLS) regression, a technique that derives latent variables with maximal covariance between sets of data, in the Rockland sample to examine age related trends in an executive function battery, the Delis–Kaplan Executive Function System (D-KEFS), which include 9 standardized tests: trail making, verbal fluency, design fluency, color-word interference, sorting, 20 questions, word context, tower, and proverb tests. The age matrix was comprised of a polynomial expansion of age, up to a 3rd degree polynomial, while the D-KEFS matrix incorporated the 18 variables from the following sub-tests: trail-making, verbal fluency, design fluency, color-word interference (the Stroop task), sorting, 20 questions, word context, tower, and proverb tests. This procedure generated 3 latent executive function factors. We examined the relationship between each factor and the integrity of the caudate M-L and diagonal connectivity gradients.

## Results

**Figure 2:**
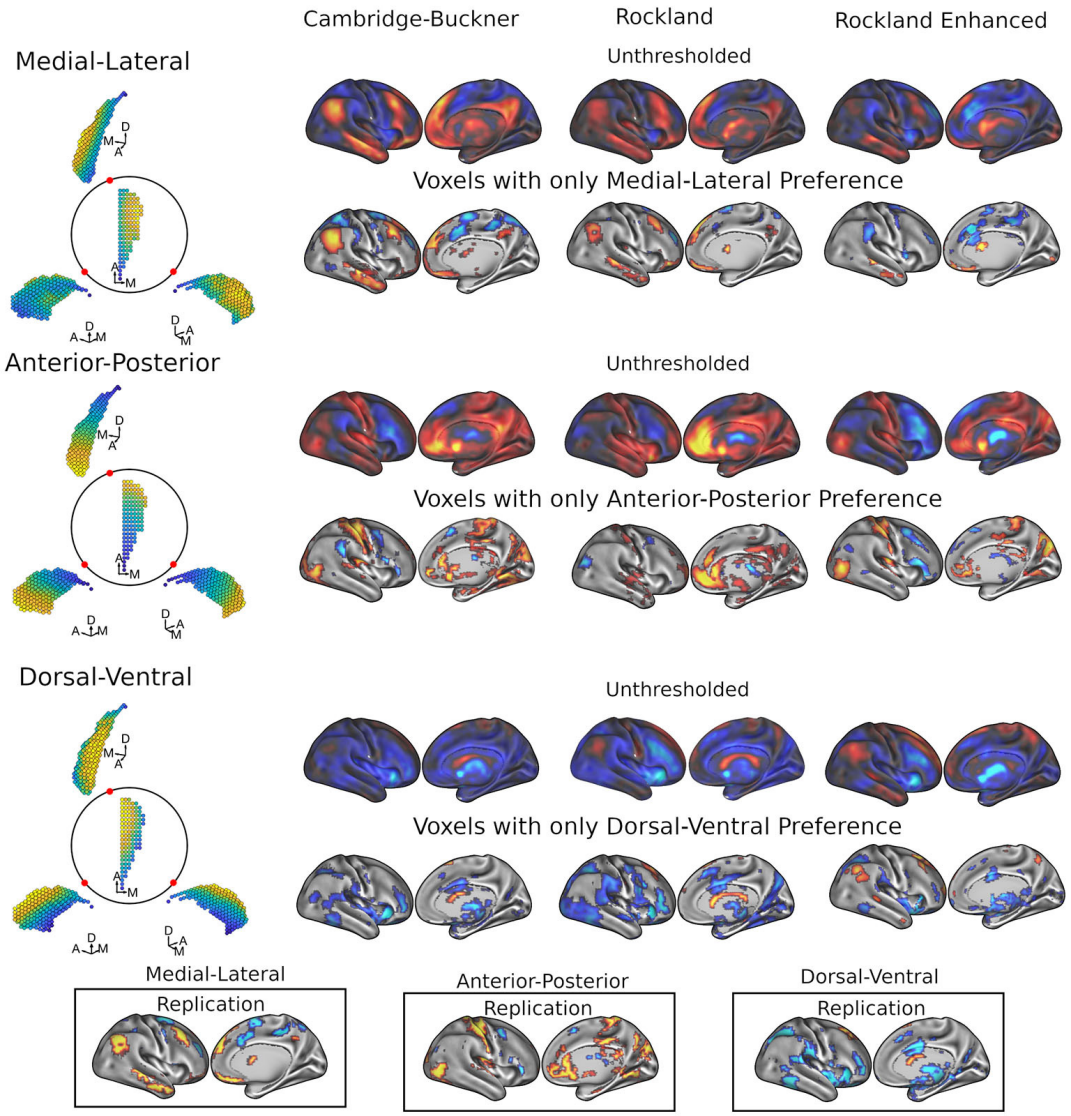
Results of FiRST analysis on caudate functional connectivity across 3 datasets: Cambridge-Buckner, Rockland, and Rockland Enhanced. For each data sample, we calculated the spatial betas uniquely related to each of the three spatial dimensions of caudate, which are orthogonalized: M-L, A-P, and D-V. For each caudate gradient (illustrated on the left from different viewpoints of the right caudate), the unthresholded (first rows) and thresholded (second rows) maps show the associated cortical areas while controlling for the other two dimensions. In the bottom panels, we calculated the replication maps across the three data samples, where a voxel was maintained if it surpassed threshold in 2/3 data samples. Color code: warmer colors represent more medial, anterior, and dorsal connectivity, whereas cooler colors represent more lateral, posterior, and ventral connectivity with the caudate for the M-L, A-P, and D-V axis, respectively.

We used a spatial regression approach to demonstrate that the functional connectivity patterns of caudate with the rest of the brain are dependent on its own spatial geometry (see Figure 1). For each voxel in the whole brain, this analysis generated an estimate of the slope of the functional connectivity along each of the three orthogonal axes of the caudate nucleus. Using three datasets of a combined total 686 subjects (198, 189, and 299), we found reliable patterns of preferred functional connectivity gradient across the geometry of the caudate (Figure 2). As both left and right caudate analyses yielded similar results, the results and discussion are described only for the right caudate for simplicity.

### Gradient connectivity along three main axes

#### Medial-lateral gradient of caudate connectivity

Figure 2 (top two rows) shows the whole-brain maps for the right caudate's M-L functional connectivity gradient. The medial extent (warmer colors) of caudate demonstrated preferential connectivity with the dorsomedial and ventromedial prefrontal cortex (dmPFC and vmPFC), along with the posterior cingulate cortex/precuneus (PCC/Pcu), angular gyrus, and middle temporal gyrus; these regions are commonly referred to as part of the default mode network (DMN) (Raichle et al., 2001; Greicius et al., 2003). Frontoparietal network areas including the inferior frontal gyrus/frontopolar cortex, posterior dorsal portion of the middle frontal gyrus and cerebellum also showed preferential connectivity to the medial extent of caudate. The lateral extent (cooler colors) of the caudate demonstrated preferential connectivity for the middle frontal gyrus (more anterior), superior frontal sulcus/dorsal aspect of frontal eye fields (FEF), frontal operculum, supplemental motor area (SMA) extending to pre-SMA, premotor, intraparietal sulcus, and superior parietal lobule, with some of these frontal and parietal regions commonly considered as parts of the dorsal and ventral attention networks (Fox et al., 2006; Yeo et al., 2011). Lateral connectivity preference was also found with regions of the cerebellum and putamen. See Supplementary Table 1 for the coordinates of all clusters.

**Figure 3:**
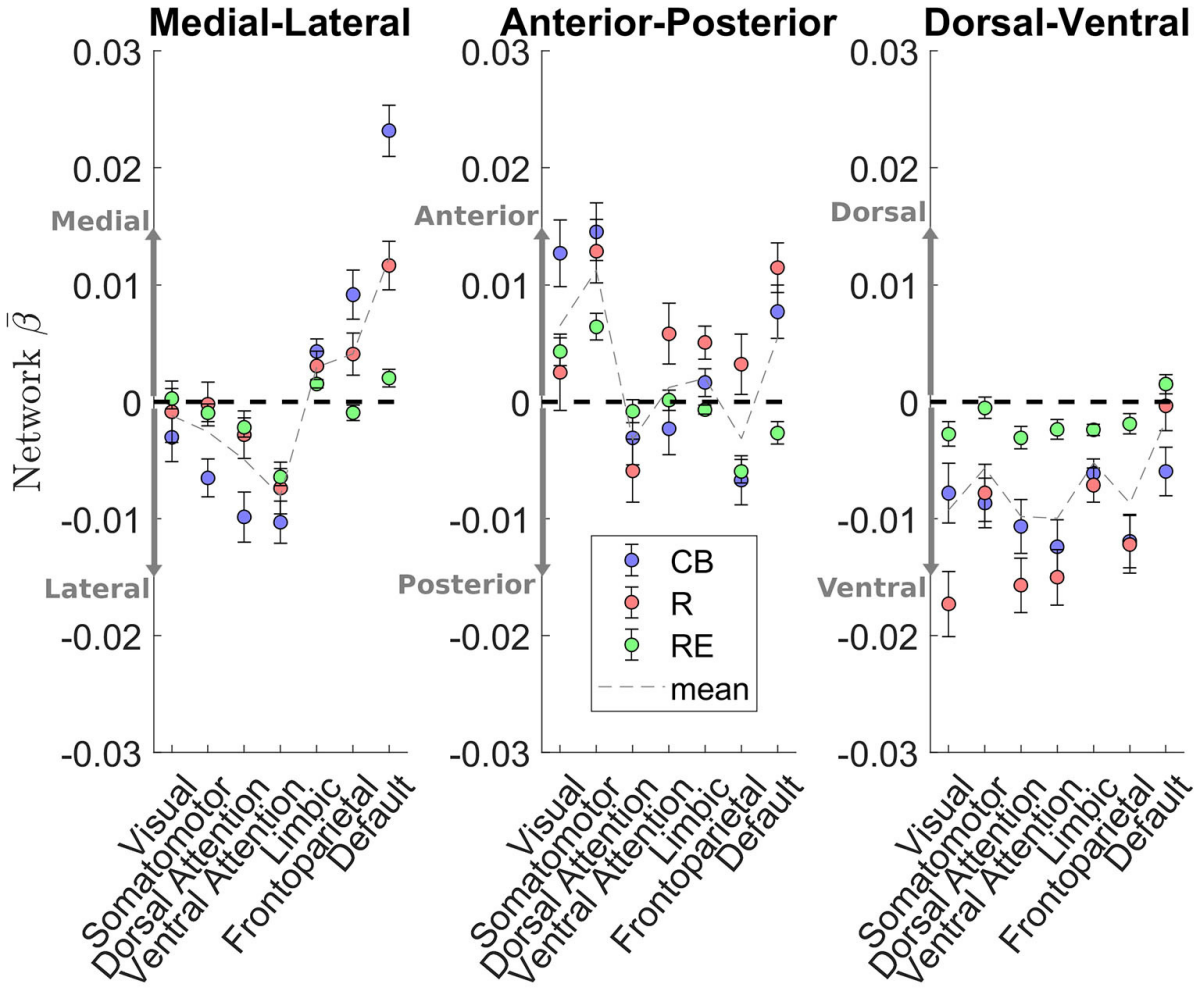
Cortical network estimation of caudate connectivity gradient along the M-L, A-P, and D-V dimensions. The medial-lateral axis shows a clear distinction between default mode network preferring to connect along the medial aspect of the caudate, while the dorsal and ventral attention networks preferred to connect along the lateral aspect of the caudate. Visual and somatomotor networks seems preferentially connect to the anterior aspect of the caudate. The dorsal-ventral gradient does not show clear patterns of connectivity with the cortical networks.

As these caudate gradient maps revealed network-level organization in line with the literature (e.g., Choi et al., 2012; Janssen et al., 2015), we conducted quantification of the M-L gradient connectivity at the network level using the 7-network partition from the literature (Yeo et al., 2011) (see Figure 3, left). Specifically, dorsal attention [Cambridge Buckner: *t*_(197)_ = −4.60, p < .001; Rockland: *t*_(188)_ = −1.39, *p* = 0.17; Rockland Enhanced: *t*_(298)_ = −2.66, *p* < 0.01] and ventral attention [*t*_(197)_ = −5.66, *p* < 0.001; *t*_(188)_ = −3.33, *p* = 0.001; *t*_(298)_ = −8.93, *p* < 0.001] networks demonstrated significant connectivity preference with the lateral caudate, while limbic [*t*_(197)_ = 3.99, *p* < 0.001; *t*_(188)_ = 2.45, *p* < 0.05; *t*_(298)_ = 4.21, *p* < 0.001] and default mode network [*t*_(197)_ = 10.52, *p* < 0.001; *t*_(188)_ = 5.60, *p* < 0.001; *t*_(298)_ = 2.71, *p* < 0.01] demonstrated significant connectivity preference with the medial caudate. The M-L gradients were less consistent for the frontoparietal network across the datasets, whereas the visual and motor networks did not show a strong M-L preference.

#### Anterior-posterior gradient of caudate connectivity

A different pattern of functional connectivity gradient was observed for the caudate's A-P axis (Figure 2, middle two rows). The anterior extent of caudate showed preferential connectivity with areas of sensorimotor, auditory, and visual cortices, along with pulvinar, posterior insular, precuneus, hippocampus, vmPFC/anterior cingulate, and superior frontal sulcus. More posterior portions of caudate displayed preferential connectivity with the posterior middle frontal gyrus, pre-SMA, and cerebellum (see Supplementary Table 2 for all clusters). Quantification at the network level is shown in Figure 3 (middle). Specifically, visual [*t*_(197)_ = 4.43, *p* < 0.001; *t*_(188)_ = 0.77, *p* = 0.44; *t*_(298)_ = 3.60, *p* < 0.001] and sensorimotor [*t*_(197)_ = 5.93, *p* < 0.001; *t*_(188)_ = 4.71, *p* < 0.001; *t*_(298)_ = 5.67, *p* < 0.001] demonstrate significant connectivity preference with the anterior extent of caudate, while dorsal attention network shows weak connectivity preference with the posterior extent of caudate [*t*_(197)_ = −1.34, *p* = 0.18; *t*_(189)_ = −2.20, *p* < 0.05, *t*_(298)_ = −0.84, *p* = 0.40]. The A-P gradient preference was less consistent for the default mode and frontoparietal networks across the datasets, whereas the other networks did not show a strong preference.

#### Dorsal-ventral gradient of caudate connectivity

Unlike the two other gradients, the D-V gradient did not show clear biologically plausible patterns (Figure 2, lower two rows). The more dorsal portions of caudate showed some connectivity preference with the superior frontal gyrus, but along with voxels mostly in the ventricles and white matter. The more ventral portions of the caudate appeared to show some connectivity preference with across the large spans of cortex (see Supplementary Table 3 for all clusters). Supporting the interpretation of this gradient simply segregating areas of meaningful BOLD signal and areas of noise BOLD signal, the network extractions demonstrated a ventral preference for every network in the brain (Figure 3, right).

### Age, integrity of caudate topography, and cognitive decline

**Figure 4:**
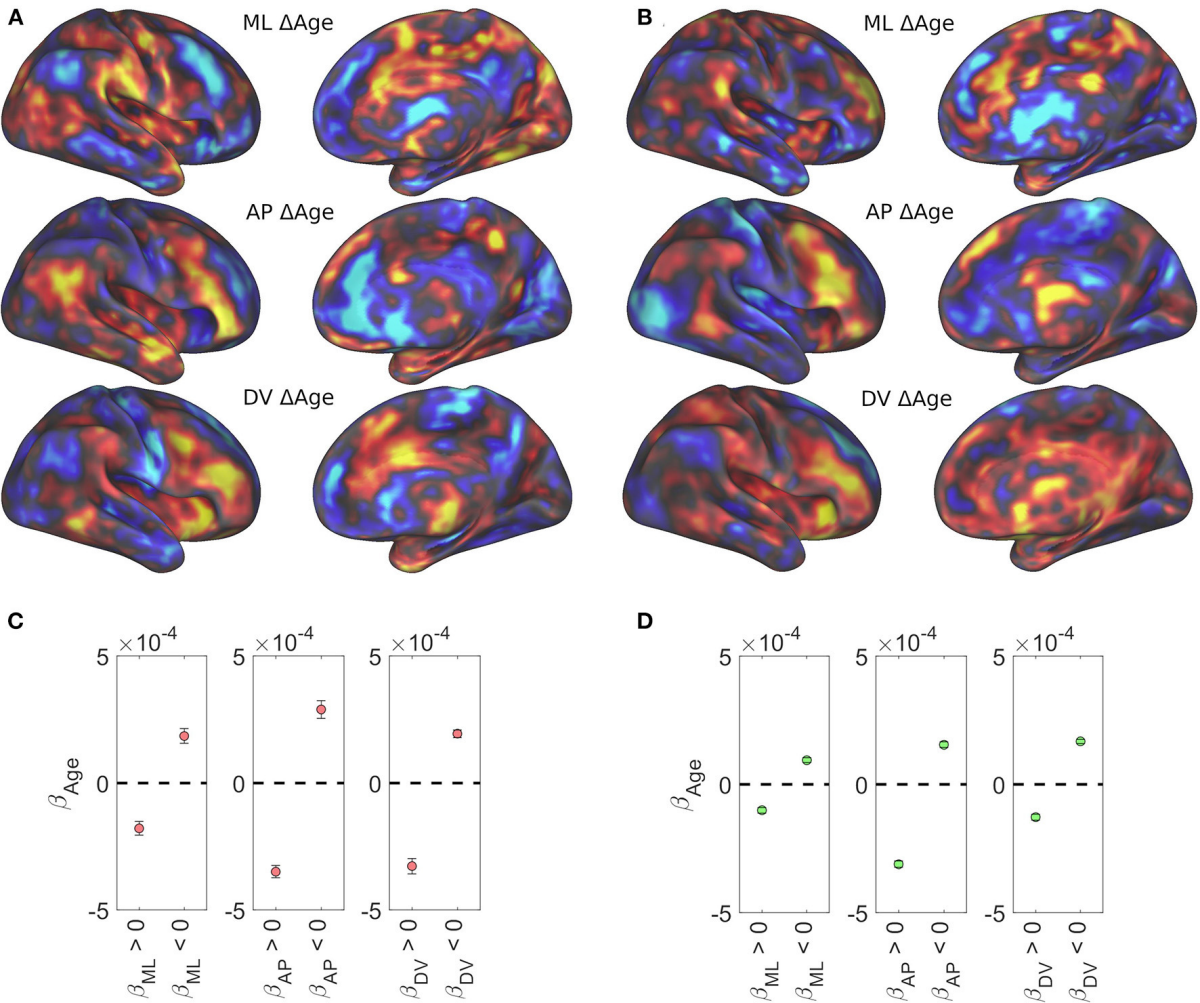
Age-related regression results. All three caudate functional gradients showed reversed association with age. Age-related regression results for the Rockland sample **(A)** and the Rockland Enhanced sample **(B)** are shown unthresholded across the brain. Average beta weight for age across significantly positive and significantly negative spatial weights in the Rockland sample **(C)** and the Rockland Enhanced sample **(D)**.

Age-related changes in caudate volume and connectivity have been shown and implicated in neurodegeneration and age-related decline in cognitive performance (Verstynen et al., 2012b; Baggio et al., 2015; Huang et al., 2017; Rieckmann et al., 2018). We examined the relationship between age and caudate functional organization using the Rockland and Rockland Enhanced samples as they have a wide age range (4–85 years). Figures 4A,B shows age related effects across the three spatial gradients of the caudate nucleus. Specifically, these data showed that the spatial distribution of age-related beta-weights were inversely related to the gradient weights (color reversed in comparison with Figure 2, top row), suggesting degradation of caudate functional organization with age (both positive and negative weights trend toward zero). To evaluate this observation quantitatively, we selected voxels based on each dataset's disjunction map, producing average age-related betas from voxels that have a positive or negative spatial gradient beta (Figures 4C,D). Universally, across datasets and spatial gradients, the age-related beta average is in the opposite direction of the gradient beta (i.e., negative for medial bias and positive for lateral bias).

**Figure 5:**
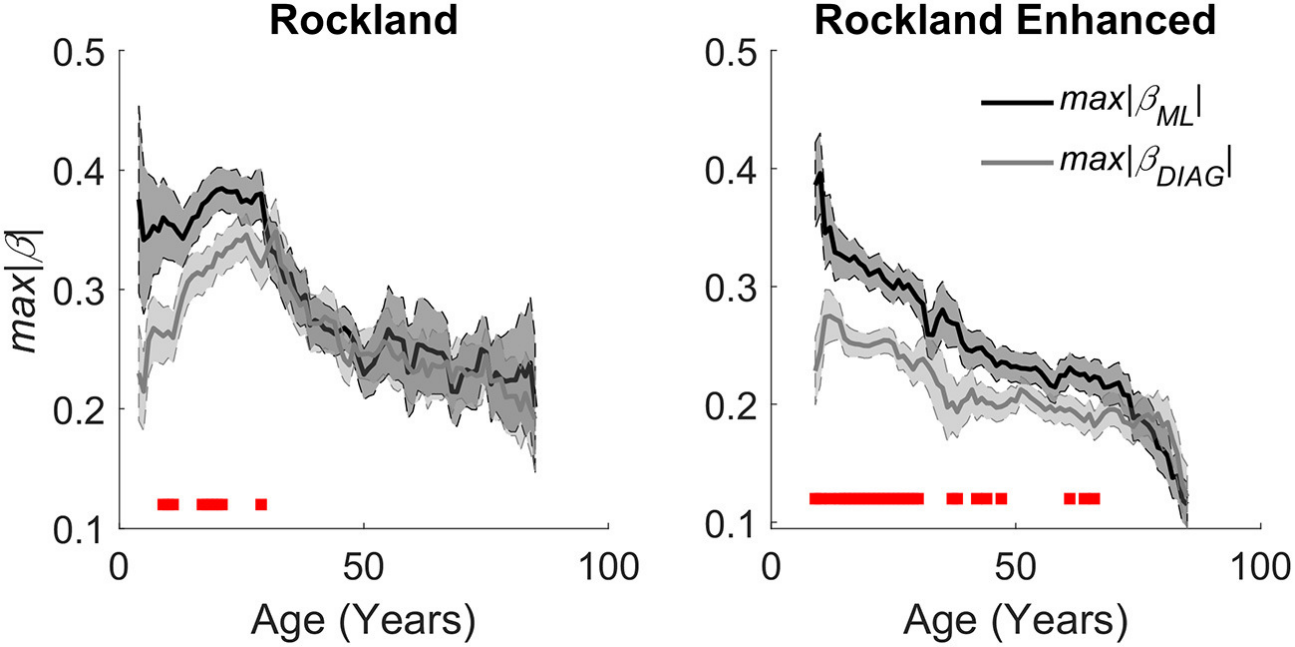
Aging effects on quantitative estimation of the integrity of caudate gradients. **(Left)** Caudate spatial integrity estimates across age groups using a sliding window mean of 14 years of the Rockland data sample. **(Right)** Replication of aging effects in the Rockland Enhanced data sample. Red bars at the bottom represent a significant difference between the two gradients with an alpha of .05, demonstrating that there is a bias in organization across M-L early in age that evens out as the organization degrades with age.

Further, we used a new metric to compute the integrity of each individual's caudate functional topology to relate to age and examined to what extent that their caudate's gradient integrity contributes to age related changes in executive function. We used two gradient templates estimated in previous work (O'Rawe et al., 2019), medial-lateral (related to the M-L axis here) and diagonal (related to the A-P axis here) gradients, which showed similar cortical connectivity maps as the M-L and A-P gradients described above and shown in Figure 2 (see Supplementary Figure S1). The fit of each subject's estimated caudate gradients with the templates was used as a measure of the integrity of their caudate's connectivity gradient (see Methods). We found a clear negative correlation with older age for both gradients {ML: [*r*_(187)_ = −0.34, *p* < 0.001; *r*_(297)_ = −0.37, *p* < 0.001], Diagonal (or A-P): [*r*_(187)_ = −0.20, *p* < 0.01; *r*_(297)_ = −0.25, *p* < 0.001]} (Figure 5).

**Figure 6:**
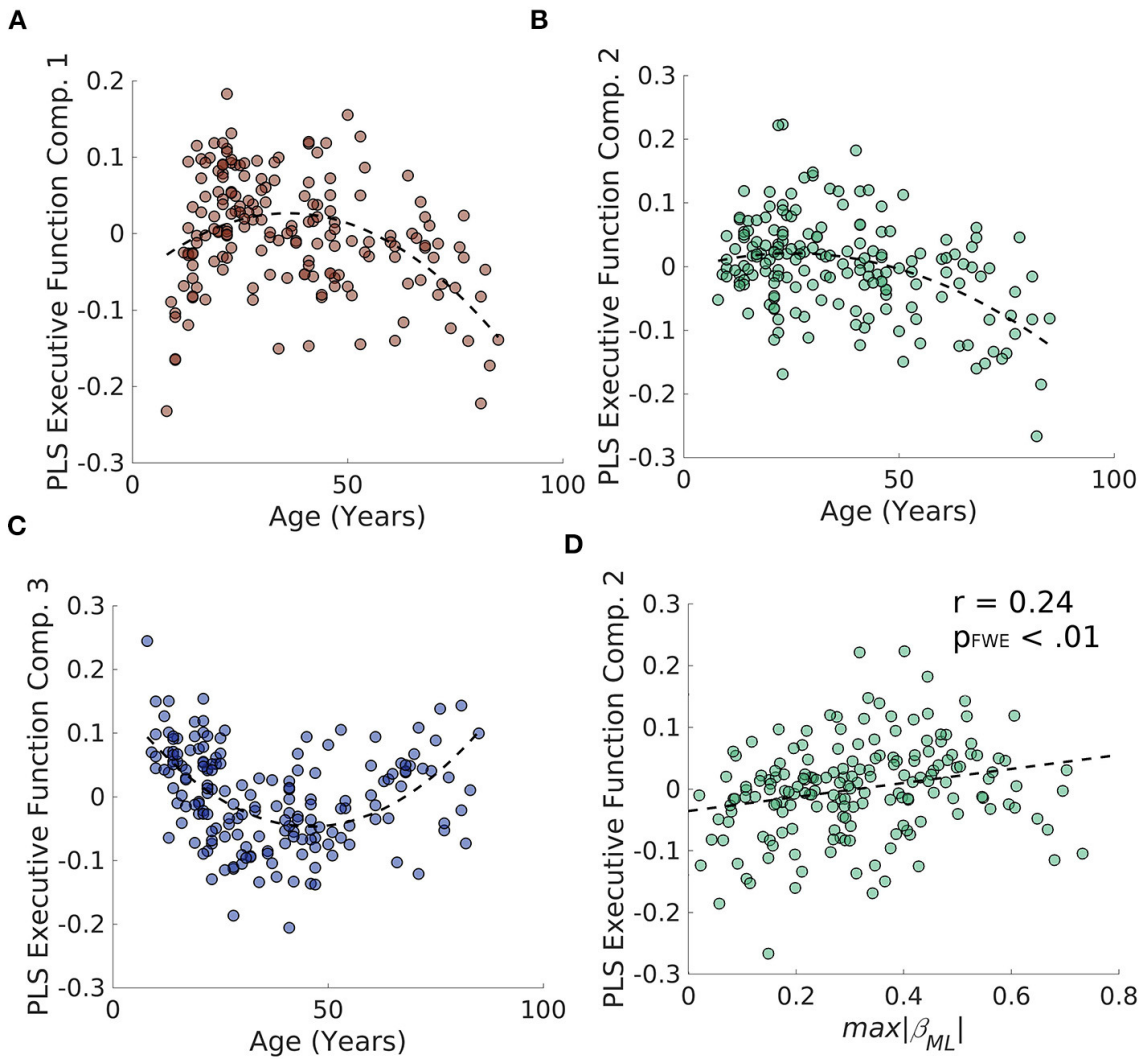
Results of PLS regression of age polynomial expansion (up to 3rd order) on the D-KEFS inventory for the NKI/Rockland sample. **(A–C)** The three age-related latent executive function variables plotted across age. **(D)** The second age-related latent executive function variable significantly correlates with the integrity of the M-L gradient in the caudate.

To examine whether degradation of the caudate's connectivity gradient is related to aging-related decline in verbal and non-verbal executive functions, we performed a partial least squares (PLS) regression on the D-KEFS test scores and a 3rd order polynomial expansion of age in the Rockland sample and extracted 3 age-related latent executive function variables (Figures 6A–C; Supplementary Figure S2 for loadings). We then examined whether these 3 latent variables were related to estimates of caudate's M-L or A-P gradient integrity and found that the only relationship that surpassed FWE thresholds was the relationship between M-L gradient integrity and the PLS component 2 [*r*_(187)_ = 0.24, *p*_FWE_ < 0.01] (Figure 6D). This relationship was in part driven by the age-related variance, as including age as a regressor in a linear model reduced the effect size to half its original size (β = 0.12, *p* = 0.12).

## Discussion

The cortico-striatal network topology is postulated to support hierarchical behavioral control processes from sensorimotor response selection to more abstract cognitive control (Alexander et al., 1986; Frank and Badre, 2012). Damage to these circuits has been shown to produce various cognitive changes in healthy development and aging, movement disorders, and a wide range of psychiatry illnesses (DeLong and Wichmann, 2007; Leisman et al., 2014; Gunaydin and Kreitzer, 2016). Previous studies of structural and functional connectivity have shown that the topographic organization of the human striatum resembles that of the non-human primates (Jarbo and Verstynen, 2015; Marquand et al., 2017; O'Rawe et al., 2019; Liu et al., 2021). However, most fMRI studies have suggested a dominant rostral-caudal functional organization across the entire striatum (see review by Vogelsang and D'Esposito, 2018), even though a global medial-lateral gradient within the caudate nucleus (Jarbo and Verstynen, 2015; O'Rawe et al., 2019) and more complex functional organizations (Jarbo and Verstynen, 2015; Choi et al., 2018) have been observed. Since most fMRI studies treated the putamen and the caudate nucleus as a single striatal system, the organizational layout of the cortico-caudate circuits in the human brain remains unclear, especially at the single subject level. Here, we used a method that estimates the continuous spatial organization of the caudate's functional connectivity with the rest of the brain and provide a metric that indexes the integrity of the caudate's functional topology at the individual subject level and related it to age-related changes in cognitive control.

### Two organizational principles within the caudate nucleus

Utilizing the continuous space provided by resting-state fMRI data, we found a stable functional organization of the cortico-caudate topography along both A-P and M-L axes (but not D-V) of the caudate nucleus (Figure 2). This observation corroborates early anatomical tracing studies of non-human primates (Yeterian and Van Hoesen, 1978; Selemon and Goldman-Rakic, 1985; Parthasarathy et al., 1992) and confirms that there are at least two organization principles within the human caudate (O'Rawe et al., 2019). Similar findings were reported in previous human neuroimaging studies using more conventional voxel-based connectivity analysis (Choi et al., 2012) and diffusion tensor imaging with tractography (Jarbo and Verstynen, 2015). Our analysis, however, revealed that these caudate topographic organizations, particularly the longitudinal zones along the M-L axis, showed more reliable distinctive associations with the large-scale, canonical cortical networks (Figure 3) across all three datasets. Our findings further suggest that the M-L caudate gradient seems to break down more across age, which is potentially meaningful to age related changes in cognitive control of behavior (Figures 4–6). Indeed, the caudate was long posited as a central component of higher order neural circuits for supporting action selection (Mink, 1996; Grahn et al., 2008), Our findings suggest that the M-L functional organization of cortical-caudate connectivity may support executive functions that is shown to be sensitive to aging (Huang et al., 2017). We also provided an index to quantify the integrity of caudate functional organization that can be used to relate to individual differences in behavioral outcomes.

Some differences in functional organization seem evident between our findings and previous findings. For example, we observed that some parts of the frontal and parietal cortices are functionally coupled with the more posterior extent of the caudate. This seems different from the original A-P or rostral-caudal gradient shown in previous studies of animals using anatomical tracing (e.g., Kemp and Powell) and humans using fiber tractography (e.g., Draganski et al., 2008; Verstynen et al., 2012a), although these previous analyses were more restricted to local organizations in the striatum. It is possible that these apparent discrepancies may reflect potential differences between structural connectivity and functional connectivity in human imaging data. However, the differences more likely originate from aiming to parcellate the striatum into discrete units vs. mapping through the continuous anatomical space. Nonetheless, our finding is in agreement with more recent DTI findings by Jarbo and Verstynen (2015), which also showed a global medial-lateral gradient but a more local A-P gradient in both DTI tractography and resting-state fMRI functional connectivity data.

### The medial-lateral topographic organization implications for cognitive control of behavior and aging

Our findings highlight the potential importance of the M-L functional topography of the caudate nucleus in the human brain. We showed that the medial caudate seems to be differentially connected to default mode and limbic networks, while the lateral caudate seems to be differentially connected to the dorsal and ventral attention networks. These findings are consistent with the anatomical tracing literature in macaques, which showed that the medial caudate receives projections from orbitofrontal cortex, vmPFC, dmPFC, and superior temporal gyrus (STG) (Yeterian and Van Hoesen, 1978; Selemon and Goldman-Rakic, 1985; Yeterian and Pandya, 1991; Ferry et al., 2000), while the lateral caudate receives projections from intraparietal sulcus (IPS), inferior parietal lobule (IPL), frontal eye fields (FEF), and supplementary eye fields (SEF) (Selemon and Goldman-Rakic, 1985; Parthasarathy et al., 1992). It has been, however, unclear what exact functions, if any, this organization provides until recently. Morris et al. (2022) have recently found that the caudate constrains the effect of prediction error on causal inference by the observed covariances in the environment, *via* interactions with parietal lobe and medial prefrontal cortex. Our findings suggest that the medial-lateral organization may provide the anatomical framework for the integration of prediction error and observed environmental covariances.

Alternately, the M-L associated network segregation may delineate processes depending on internal and external information, as previous literature has implicated default mode/limbic networks in controlling internally guided decisions and implicated dorsal/ventral attention networks controlling externally guided decisions (Nakao et al., 2012). The striatum organizing inputs of these networks along the M-L axis may provide a substrate for shifting between these two strategies. Given a recent literature of systematic modeling of reinforcement learning within complex environments (Niv et al., 2015; Leong et al., 2017), it is possible that the caudate is integrating and leveraging information from attention deployment and from internally learned rules. Interestingly, shifts in attention during reinforcement learning in complex environments correlates to increased activity in the networks identified as more related to lateral caudate, and the maintenance of values during this process correlates with activity in networks more related to the medial caudate (Niv et al., 2015; Leong et al., 2017).

Within these models, older adults show a deficit in reinforcement learning, and they potentially compensate for this *via* increased selective attention to fewer environmental features (Radulescu et al., 2016), which is supported by our data suggesting a reduction in the caudate's M-L functional topology across age. This interpretation also is consistent with the interpretation that medial-lateral sweeps of dopamine function as spatially specific credit assignment, reinforcing circuits recently leveraged for successful behavior. In other words, this medial-lateral dopamine sweep is potentially overlaid on top of existing spatially discrete circuits along the dorsomeidal to dorsolateral gradient (Hamid et al., 2021).

### Limitations

One major difference between our gradient mapping approach from others is that we used a linear fitting of connectivity across the anatomical space instead of a non-linear fitting like that used by Haak et al. (2017). We chose the linear transformation approach as the majority of the topographic maps shown by previous anatomical tracing studies are described along the three orthogonal axes of the striatum. Since converging zones within caudate have been observed (e.g., Jarbo and Verstynen, 2015; Choi et al., 2018), future studies should implement both linear and non-linear transformations to reveal the full complexity of the cortico-striatal topology and distinguish their potential behavioral relevance. Aging-related brain atrophy may affect the estimation of functional gradient measures across age, which is an important issue in studying age-related changes functional organization. However, it is unlikely to affect our main findings as age-related effects seem evident starting from young adulthood to older ages.

## Summary

In sum, we have demonstrated both an A-P and M-L organization of the caudate functional connectivity to the rest of the brain in three independent samples of human subjects. Our findings reveal systems level of functional organization involving the caudate and that this organization degrades with age in correspondence with a reduction in executive functions. Future studies should further examine to what extent the M-L caudate gradient is linked to the more specific default and frontoparietal subnetworks that was identified at the individual subject level (Braga and Buckner, 2017). Future studies will also be necessary to understand the implications of such intrinsic striatal organizations in individual differences in cognitive control of behavior, and how much of it is dependent on dopaminergic input.

## Data availability statement

Data used for this study were obtained from the following links: Cambridge Buckner subset, http://fcon_1000.projects.nitrc.org; NKI/Rockland data sample, http://fcon_1000.projects.nitrc.org/indi/pro/nki.html; and Rockland Enhanced data sample, http://fcon_1000.projects.nitrc.org/indi/pro/nki.html.

## Ethics statement

Ethical review and approval was not required for publicly available data. Written informed consent to participate in this study was provided in the original contributions presented in the study.

## Author contributions

JO'R processed and analyzed the data and wrote the initial draft. H-CL edited the manuscript and revised the manuscript for final publication. All authors designed the research. All authors contributed to the article and approved the submitted version.

## Funding

This work was supported by the Stony Brook Research Foundation.

## Conflict of interest

The authors declare that the research was conducted in the absence of any commercial or financial relationships that could be construed as a potential conflict of interest.

## Publisher's note

All claims expressed in this article are solely those of the authors and do not necessarily represent those of their affiliated organizations, or those of the publisher, the editors and the reviewers. Any product that may be evaluated in this article, or claim that may be made by its manufacturer, is not guaranteed or endorsed by the publisher.
