# Supplementary material for: Topographic organization of the human caudate functional connectivity and age-related changes with resting-state fMRI
Source: Front Syst Neurosci. 2022 Sep 23;16:966433. doi: 10.3389/fnsys.2022.966433 (PMC9543452; doi:10.3389/fnsys.2022.966433)
Supplement: Supplementary file 1 [file Data_Sheet_1.docx]

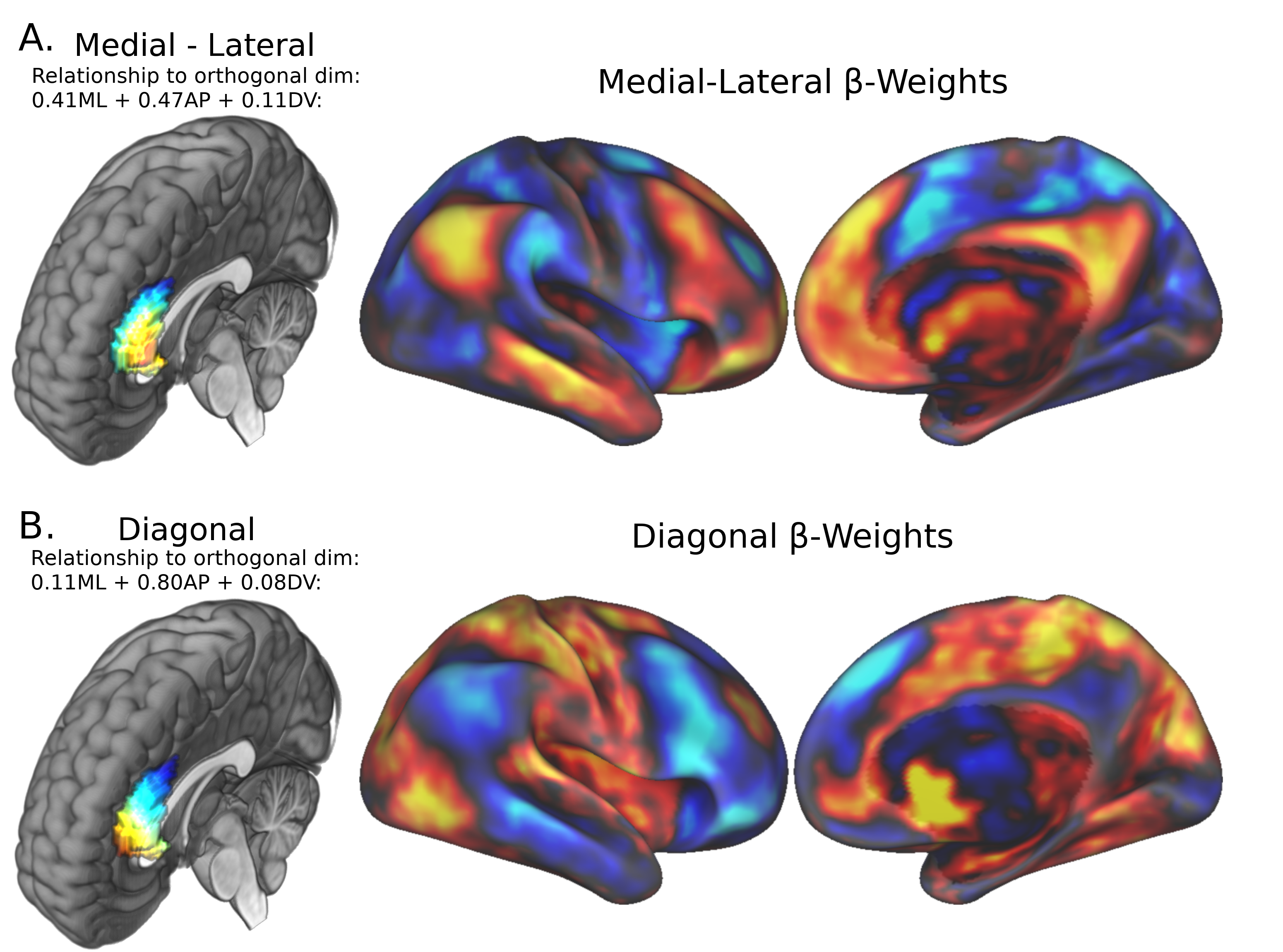


Figure S1: Caudate connectivity gradients derived using prior templates. Medial-lateral gradient **(A)** and Diagonal gradient **(B)**, as defined by previous empirically driven gradient discovery across the striatum (O’Rawe, Ide, and Leung, 2019), demonstrate similar caudate-cortical biases as the orthogonal M-L and A-P dimensions shown in Figure 2. Note: For both (A) and (B), the image on the left shows the caudate spatial gradient in the right hemisphere and the two images on the right are the corresponding caudate-cortical connectivity maps.


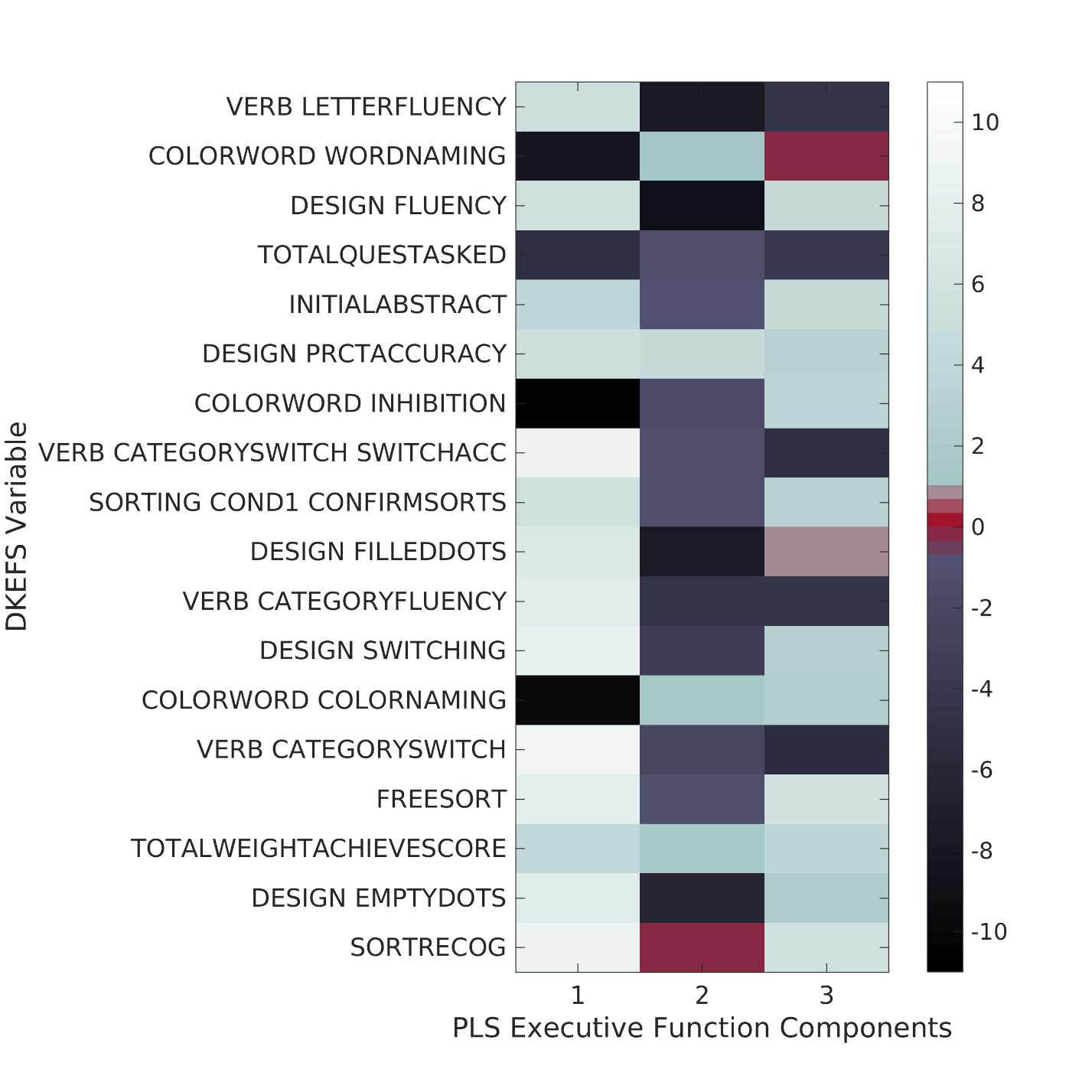
Figure S2: D-KEFS PLS loading matrix. Brighter colors denote larger positive loadings, darker colors denote larger negative loading. Note, positive PLS weights for Component 1 suggest a dependence on switching, composed of both verbal and visuospatial tasks, while the negative PLS weights for Component 3 seem more related to switching in the verbal domain.
