# Supplementary material for: Topographic organization of the human caudate functional connectivity and age-related changes with resting-state fMRI
Source: Front Syst Neurosci. 2022 Sep 23;16:966433. doi: 10.3389/fnsys.2022.966433 (PMC9543452; doi:10.3389/fnsys.2022.966433)
Supplement: Supplementary file 2 [file Data_Sheet_2.pdf]

Supplemental Table 1: Medial-lateral clusters surviving replication threshold with a cluster extent k > 9 (Figure 2 bottom-most row; left)

Region Name (AAL)	MNI (X)	MNI (Y)	MNI (Z)	Cluster Size (voxels)
**Medial preferred**				
Frontal_Sup_Medial_L	1	50	41	1496
Temporal_Mid_L	-59	-26	-11	666
Angular_L	-42	-63	35	556
Angular_R	51	-60	33	499
Rectus_L	1	45	-19	470
Cerebellum_Crus2_L	-26	-80	-35	469
Cerebellum_Crus1_R	29	-79	-35	300
Frontal_Mid_2_R	40	20	43	284
Temporal_Inf_R	57	-6	-28	193
Temporal_Mid_R	66	-30	-6	187
Frontal_Mid_2_L	-35	16	37	74
Cerebellum_9_L	0	-54	-47	58
Frontal_Mid_2_R	39	56	-12	29
Frontal_Mid_2_L	-38	55	-6	25
Temporal_Pole_Mid_L	-46	15	-33	15
**Lateral preferred**				
Frontal_Sup_2_R	6	1	63	828
Frontal_Mid_2_L	-36	44	26	355
Frontal_Mid_2_R	36	46	28	193
Precuneus_L	-1	-62	63	136
Cingulate_Mid_R	15	-38	45	93
Insula_L	-44	10	1	86
Frontal_Inf_Oper_R	46	12	2	30
Cerebellum_6_L	-34	-54	-30	22
Cerebellum_Crus1_R	45	-51	-33	19
Putamen_R	24	12	2	17
Vermis_8	0	-66	-33	10
Cerebellum_8_L	-33	-48	-49	9
Precuneus_L	-11	-66	52	9

Supplemental Table 2: Anterior-posterior clusters surviving replication threshold with a cluster extent k > 9 (Figure 2 bottom-most row; middle)

Region Name (AAL)	MNI (X)	MNI (Y)	MNI (Z)	Cluster Size
**Anterior preferred**				
Postcentral_L	0	-26	59	2979
ACC_sub_L	0	36	0	1419
Cuneus_L	2	-77	32	879
Temporal_Mid_R	46	-72	-1	208
Occipital_Mid_L	-41	-76	-1	165
Lingual_R	13	-57	-3	126
Lingual_L	-11	-60	-3	91
Rolandic_Oper_R	42	-12	17	47
Cingulate_Post_R	6	-42	21	23
Temporal_Sup_R	62	-11	7	16
Rolandic_Oper_L	-62	-9	9	12
**Posterior preferred**				
White Matter	-22	-23	20	258
White Matter	27	-16	21	255
Frontal_Mid_2_L	-37	5	56	153
Frontal_Mid_2_R	45	6	50	107
Supp_Motor_Area_L	0	18	54	89
Frontal_Inf_Oper_L	-52	13	17	86
Vermis_7	2	-80	-20	70
Parietal_Inf_L	-55	-45	45	55
Cerebellum_6_L	-30	-69	-25	49
Frontal_Inf_Tri_R	54	34	0.5	49
Cerebellum_6_R	31	-69	-27	37
Temporal_Mid_L	-60	-55	-3	22
White Matter	0	-30	9	16
Frontal_Inf_Oper_R	55	18	16	16
White Matter	-25	6	20	14
SupraMarginal_R	63	-43	39	13
Frontal_Sup_2_R	35	4.5	64	9

Supplemental Table 3: Dorsal-ventral clusters surviving replication threshold with a cluster extent k > 9 (Figure 2 bottom-most row; right)

Region Name (AAL)	MNI (X)	MNI (Y)	MNI (Z)	Cluster size
**Dorsal preferred**				
Frontal_Sup_2_L	-15	20	60	317
White Matter	22	28	15	196
Frontal_Sup_2_R	25	27	52	188
Frontal_Sup_2_L	-18	53	30	104
Ventricle	-30	-46	9	90
Ventricle	33	-47	9	81
Frontal_Mid_2_L	-34	26	42	48
White Matter	-15	18	16	31
Frontal_Sup_2_R	18	58	27	28
Angular_L	-50	-57	35	27
Cerebellum_Crus2_R	44	-52	-48	26
Ventricle	1	1	21	26
Cerebellum_Crus1_R	50	-61	-31	19
**Ventral preferred**				
Cerebellum_7b_L	-28	-70	-48	10
Cerebellum_8_R	10	-74	-40	32
Cerebellum_Crus2_L	-6	-77	-37	21
Temporal_Pole_Mid_R	27	7	-37	21
Fusiform_L	-30	-32	-25	30
Pallidum_R	15	1	-5	4157
Temporal_Inf_L	-48	-56	-14	694
Temporal_Inf_R	52	-51	-15	451
Occipital_Mid_L	-27	-95	-5	317
Rolandic_Oper_R	61	5	3	42
Temporal_Sup_L	-59	-26	18	473
Occipital_Mid_L	-48	-77	17	49
Insula_L	-36	-3	12	13
Occipital_Mid_L	-32	-89	26	12
Parietal_Inf_L	-30	-56	44	551
Cingulate_Mid_L	0	1	33	274
Precentral_L	-45	0	36	19
Parietal_Inf_R	33	-54	44	578
Frontal_Sup_Medial_R	5	24	42	14
